# Tensor methods in data analysis of chromatography/mass spectroscopy-based plant metabolomics

**DOI:** 10.1186/s13007-023-01105-y

**Published:** 2023-11-21

**Authors:** Lili Guo, Huiwen Yu, Yuan Li, Chenxi Zhang, Mourad Kharbach

**Affiliations:** 1 Weifang University of Science and Technology, Shouguang, 262700 China; 2 Shenzhen Hospital, Southern Medical University, Shenzhen, 518005 China; 3 Chemometrics Group, Faculty of Science, University of Copenhagen, Frederiksberg, 1958 Denmark; 4 Northwest Land and Resources Research Center, Shaanxi Normal University, Xi’an, 710062 China; 5 Department of Food and Nutrition, University of Helsinki, Helsinki, 00014 Finland; 6 Department of Computer Sciences, University of Helsinki, Helsinki, 00560 Finland

**Keywords:** Tensor methods, Data analysis, Chromatography/mass spectroscopy, Plant metabolomics, Chemometrics

## Abstract

Plant metabolomics is an important research area in plant science. Chemometrics is a useful tool for plant metabolomic data analysis and processing. Among them, high-order chemometrics represented by tensor modeling provides a new and promising technical method for the analysis of complex multi-way plant metabolomics data. This paper systematically reviews different tensor methods widely applied to the analysis of complex plant metabolomic data. The advantages and disadvantages as well as the latest methodological advances of tensor models are reviewed and summarized. At the same time, application of different tensor methods in solving plant science problems are also reviewed and discussed. The reviewed applications of tensor methods in plant metabolomics cover a wide range of important plant science topics including plant gene mutation and phenotype, plant disease and resistance, plant pharmacology and nutrition analysis, and plant products ingredient characterization and quality evaluation. It is evident from the review that tensor methods significantly promote the automated and intelligent process of plant metabolomics analysis and profoundly affect the paradigm of plant science research. To the best of our knowledge, this is the first review to systematically summarize the tensor analysis methods in plant metabolomic data analysis.

## Introduction

Metabolomics is a rapidly growing field that gains more and more attention from both industry and scientific communities. By integrating the capabilities of different disciplines such as analytical chemistry and statistics, metabolomics aims to gain a systematic understanding of quantitative changes in the level of metabolites from the biology or chemical system [1]. Plant metabolomics is a key research area in plant science and it refers to the quantitative analysis of metabolites in plant system. It is widely used as an important technology and tool for phenotyping and diagnostic analyses of plants [2]. Owing to its great potential in capturing the molecule changes from complex biological system, metabolomics technology is also used for functional annotation of genes and understanding the cellular response to biological conditions in plant science [3]. Apart from these typical applications, metabolomics technology has also been used for understanding other complex plant science problems. For example, investigating the natural variance of metabolite during the plant evolution makes it possible for precise modification and personalized customization of metabolic pathways in plants [4]. Therefore, plant metabolomics is of great potential and importance for plant science investigations.

**Fig. 1:**
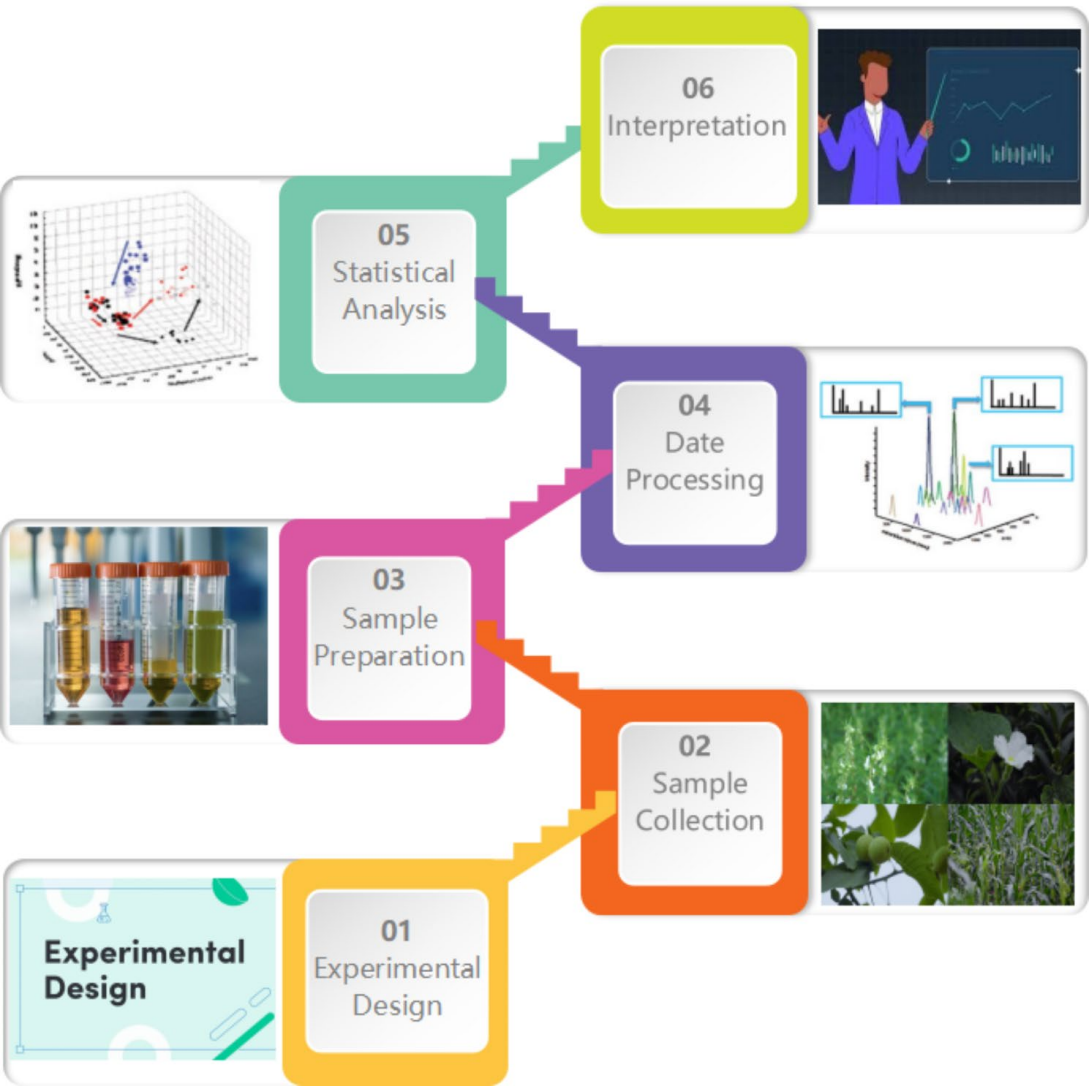
A typical plant metabolomics work flow

Plant metabolomics analysis generally falls into two categories: targeted metabolomics analysis and untargeted metabolomics analysis [5]. In targeted metabolomics analysis, the interested and specific metabolites are analyzed in a targeted way and the chemical information are selectively extracted from the whole metabolomics dataset. Unlike targeted metabolomics, in untargeted metabolomics, the analysis is generally performed in a non-specific manner. That is to say, the aim of untargeted metabolomics analysis is to extract as much metabolite’s information as possible from global metabolites spectra. The global metabolites instead of any targeted ones are of interests during the analysis [6]. Moreover, very little information needs to be known about the samples in untargeted metabolomics analysis, which makes it useful for exploratory investigations. As shown in Fig. 1, a complete plant metabolomics work flow is composed of experimental design, sample collection, sample preparation, instrumental analysis, data processing and analysis, statistical modeling and expert interpretation etc. Although the advances in instrument have been achieved in the past decades, it still remains immense challenges for constructing a more efficient and automated plant metabolomics work flow both practically and theoretically. In this review, we focus primarily on the challenges and advancements associated with data processing and analysis section in the plant metabolomics work flow, with a specific attention on tensor methods.

**Fig. 2:**
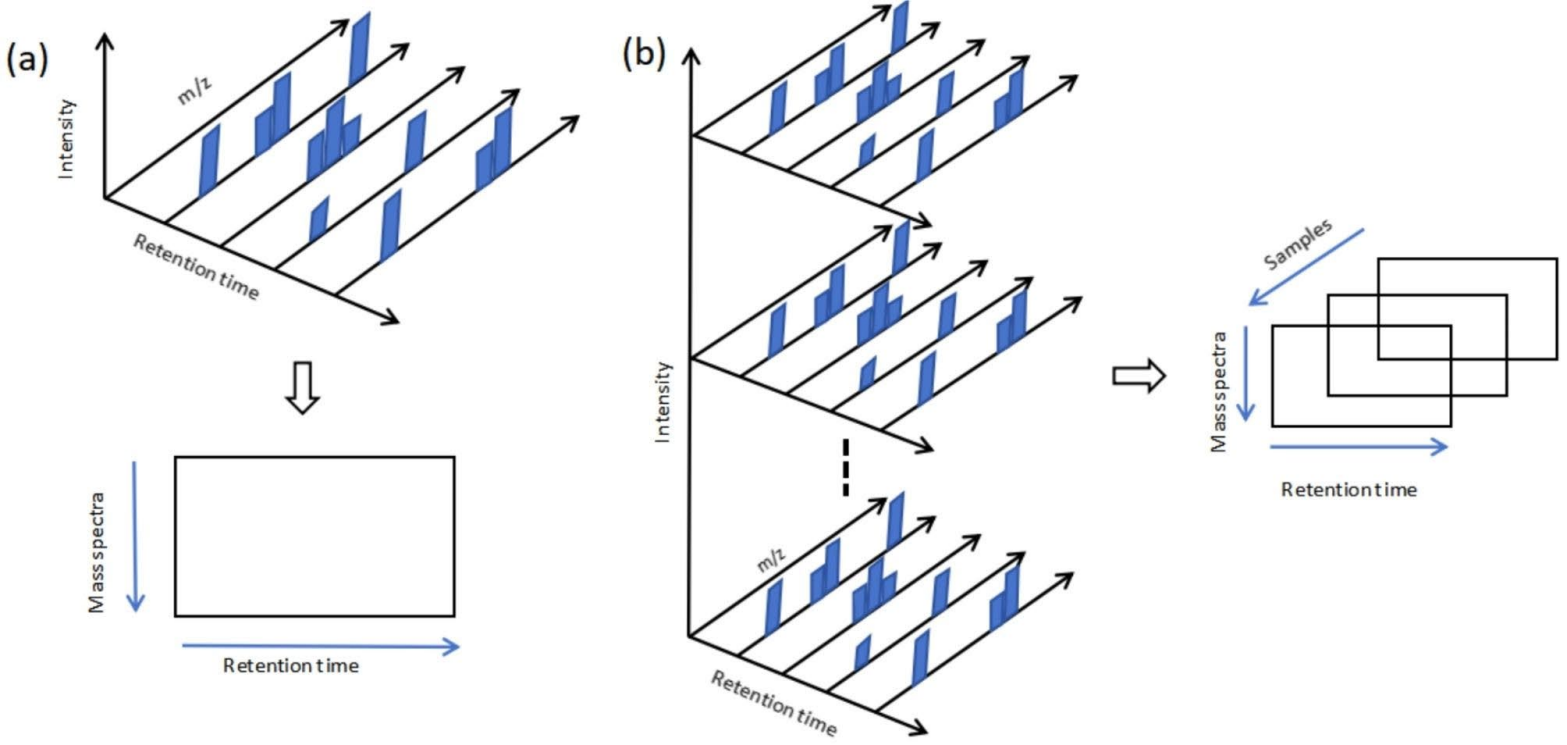
Data structure in plant metabolomics studies, taking an example of GC-MS data: (a)one sample GC-MS data-matrix. (b)multi-sample GC-MS data-tensor

Chromatography coupled with mass spectroscopy and Nuclear Magnetic Resonance (NMR) are the main types of instruments that are widely used for performing plant metabolomics analysis. NMR is a well-known technology that is capable of producing robust, reproducible and structural metabolite information when used in metabolomics studies [7]. Compared to NMR, chromatography coupled with mass spectroscopy is more advantageous in the cheaper cost, higher sensitivity and lower learning curve [8]. Gas chromatography MS (GC-MS) and liquid chromatography MS (LC-MS) are two types of chromatography coupled mass spectroscopy instruments. The combination of chromatography with strong separation ability and MS with high sensitivity identification advantages provides powerful metabolomics analysis platforms for many plant science investigations. Chromatography can be also coupled with diode array detector (DAD), which is also frequently used in many plant metabolomics applications. The aforementioned chromatography-based plant metabolomics instruments generate massive and exceptionally complex data for the analysts. Thousands of compounds can exist in a small and short metabolites spectrum. Not only the amount of the data increases dramatically, the dimensionality of data also significantly increases. For example, in the metabolomics analysis with GC-MS, the resulted data with a number of runs are organized in a three-way structure, so-called tensor structure. The three dimensions of GC-MS tensor data are named as elution profiles, mass spectra and sample concentrations respectively, as shown in Fig. 2. Compared to the data matrix containing one sample GC-MS data, containing only mass spectra and elution profiles information, the three-way GC-MS data is more complex. It is challenging to fully and efficiently extract metabolites information from such multi-way data [9]. In addition, the plant metabolomics data analysis are also facing many other analytical challenges that cannot be overlooked, including but not limited to co-eluted peaks, low intensity peaks, baseline drift, background effect, retention time shift, skewed peaks etc. [10]. In recent years, the multi-dimensional chromatographic MS so-called GC-GC-MS or LC-LC-MS has gained more and more attention from analytical plant scientists. Multi-chromatogram coupled with MS is deemed to have stronger metabolites identification capability due to its advantages in higher peak capacity and resolving power. However, it is necessary to mention that the produced data from multi-chromatogram MS is also far more complex compared to the conventional GC-MS or LC-MS data, because the dimensionality of the data increases. Therefore, one of the challenges associated with plant metabolomics analysis stems from how to handle the large metabolomics data and extract the valuable information from it. Advanced chemometrics tool and statistical modeling methods are urgently required in plant metabolomics analysis.

In recent years, tensor methods, also known as multi-way models, has been proven to be a promising high-order chemometrics tool for solving or alleviating the practical and challenges of complex metabolomics data analysis [11, 12]. Tensor modeling is the emerging topic in chemometrics, as well as in many other fields including signal processing [13], biomedical informatics [14], machine learning [15], environmental analytics [16] etc. By decomposing the multi-way array into a set of high-order components, the tensor models are capable of extracting the latent information and structure from the complex multi-way data. Unlike the two-way chemometrics tools such as PCA, tensor models are used to analyze the multi-way data without destroying the intrinsic multi-way structure of the data and some of the tensor models are able to yield unique solutions with chemical meaning [17]. Compared to the two-way statistical methods, tensor methods are able to make predictions more robust in the presence of serious noise [13]. In the context of chromatography-mass spectroscopy based plant metabolomics, tensor models have been validated to greatly simply the analysis by eliminating the need for multiple algorithms and avoiding the cumbersome human-dependent data preprocessing [8]. Tensor models represented by PARAFAC2 shows great potential for solving the analytical challenges of plant metabolomics data analysis and establishing an automated and intelligent plant metabolomics work flow [10, 11, 18]. Therefore, tensor methods open new ways for turning the massive plant metabolomics data into valuable information and investigating meaningful solutions to a wide variety of plant metabolomics problems.

The rest of the review is organized as follows. Section 2 presents the widely used tensor models in plant metabolomics data analysis and discusses the advances and limitations of various tensor models. In Section 3, we briefly introduce the recent application of tensor models in plant metabolomics investigations and summarize the plant metabolomics problems that tensor models are used to solve. In the end, we conclude the review and discuss some future perspectives on plant metabolomics data analysis coupled with tensor modeling methods.

## Tensor models

### PARAFAC

**Fig. 3:**
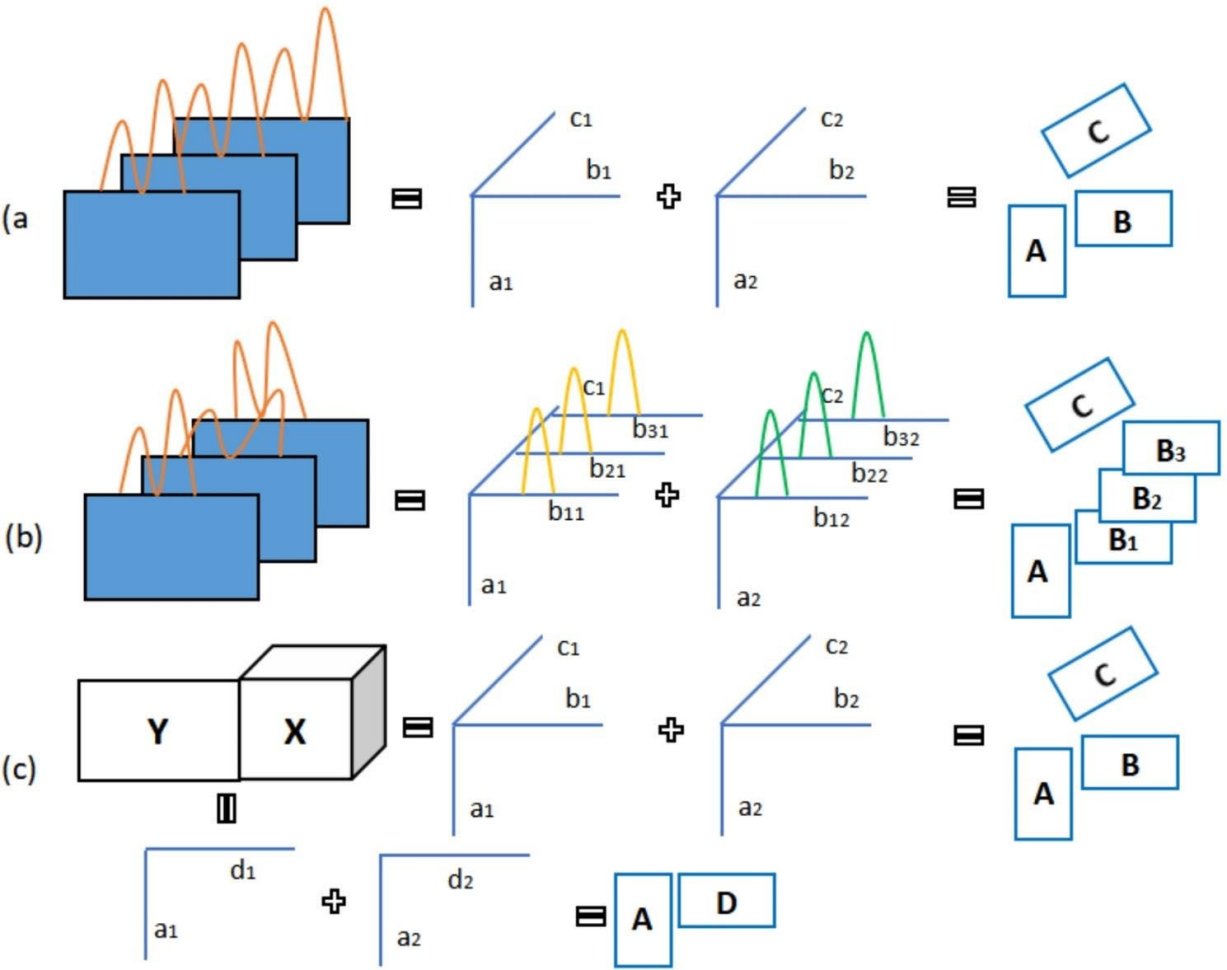
Graphical illustration of different tensor models in plant metabolomics investigations: (a)PARAFAC model. (b) PARAFAC2 model. (C) Tensor based data fusion

PARAFAC is an important tensor model for analyzing multi-way data with a multilinear structure. It was first proposed by Harshman [19], a psychometrician from Canada. In the same year, Carrol et al. proposed the same tensor model but with a different name called CANDECOMP [20]. The three-way PARAFAC model is generally written in matrix notations as follows:

$${\mathbf{X}}_{k}=\mathbf{A}{\mathbf{D}}_{k}{\left(\mathbf{B}\right)}^{\text{T}}+{\mathbf{E}}_{k}, k=1,\dots ,K \left(1\right)$$

where ${\mathbf{X}}_{k}$ is the ${k}_{th}$ submatrix (frontal slab) of the $I\times J\times K$ third-order tensor $\underset{\_}{\mathbf{X}}$, e.g., it can be the ${k}_{th}$ sample run in LC-MS metabolomics experimental analysis. The matrix ${\mathbf{E}}_{k}$ denotes the error array with a dimension of $I\times J$. For a $F$-component PARAFAC model on aligned LC-MS data, the matrix $\mathbf{A}$ ($I\times F$) may store the mass spectra, and the matrix $\mathbf{B}$ ($J\times F$) may contain the elution profile. The ${\mathbf{D}}_{k}$ ($F\times F$) is a diagonal matrix where the ${k}_{th}$ row vector of matrix $\mathbf{C}$ ($K\times F$) is a diagonal vector. The elements on the diagonal vector denote the concentration of the $f_{th}$ resolved chemical in the sample $k$. Besides the above notation, PARAFAC model can also be written in the format of Kronecker product:

$${\mathbf{X}}^{(I\times JK)}=\sum _{f=1}^{F}{\mathbf{a}}_{f}\otimes({{\mathbf{c}}_{f}}^{\varvec{T}}\otimes{{\mathbf{b}}_{f}}^{\varvec{T}})+{\mathbf{E}}^{(I\times JK)}, f=1,\dots , F \left(2\right)$$

where ${\mathbf{a}}_{f}$ is the ${f}_{th}$ column vector of matrix $\mathbf{A}$, ${\mathbf{b}}_{f}\,\text{a}\text{n}\text{d}\,{\mathbf{c}}_{f}$ are defined in the same manner. ${\mathbf{X}}^{(I\times JK)}$ is the unfolded matrix of third-order tensor $\underset{\_}{\mathbf{X}}$ (along with the second mode) and ${\mathbf{E}}^{(I\times JK)}$ is the residual array with a dimension of $I\times JK$. $\otimes{}$ is Kronecker product. A graphical illustration of PARAFAC model is presented on Fig. 3.

It is vital to note that the PARAFAC model is multilinear. In other words, if the underlying profiles change shape or shift in one mode across another mode, then such data will violate the multi-linearity assumption of PARAFAC model, thus applying PARAFAC model will not lead to chemically meaningful tensor solution. When using PARAFAC model on large scale GC-MS or LC-MS data, the data has to be preprocessed or aligned. This is because chromatographic data is always shifted run by run and such shift violates the multilinear assumption of PARAFAC model. Under mild condition [21], it is proved that the solution of the PARAFAC model is unique, which is one of the significant merits of this model. The alternating least squares (ALS) algorithm is the most widely used algorithm for fitting a PARAFAC model. In ALS, the subset of the estimated loading matrices is successively updated, and this procedure is iteratively repeated until the algorithm reaches a convergence criterion. Since the principles of ALS method are simple to understand, and many useful constraints, such as non-negativity and unimodality, are easy to impose within the algorithm, PARAFAC-ALS has been one of the most customary algorithms so far. However, many problems exist in alternating algorithms. For instance, the local minima problem is a non-trivial numerical and practical challenge [22]. Moreover, the efficiency of PARAFAC-ALS algorithm is practically low and it is also difficult to converge in the case of swamps, especially for larger datasets.

In recent years, some potential solutions have been proposed to attack the problems of alternating based PARAFAC algorithm. Zeng et al. proposed an alternating minimization-based method for incremental PARAFAC decomposition [23], and it showcased great advantages in computational time. De et al. developed an L-BFGS based accelerator for ALS and applied it on tensor decomposition [24], their results showed there were substantial improvements in terms of convergence time over the available methods. The principles of randomization have also been successfully extended and applied to alternating based PARAFAC algorithm. For example, Vervliet et al. developed a PARAFAC-ALS tensor decomposition algorithm by applying a randomized block sampling method [25]. The test results indicated the new algorithm achieved computational savings and attained near-optimal accuracy, even though it may be slow in the case of ill-conditioned situations. Erichsion et al. proposed a randomized algorithm [26]. In their method, the random projections and power iterations were employed to yield a compressed tensor and then the ALS procedure was applied to the compressed tensor. They concluded that the new algorithm significantly reduced the computational cost of CP tensor decomposition. Another group of algorithms for fitting the PARAFAC model is the derivative based algorithm. Instead of calculating the least square solution and successively updating subsets of the estimated matrices in each step, derivative based algorithms update all the parameters in each step by calculating the Jacobian and approximate Hessian matrix. The representative algorithms are PMF3 [27], damped Gauss Newton [28], low complexity damped Gauss Newton [29], inexact generalized Gauss-Newton method [30] and weighted Krylov-Levenberg-Marquardt method [31]. As one would expect, these derivative based algorithms are beneficial for the convergence in the case of swamp or ill-conditioned data, owing to their second-order advantages and the super-linearity in the vicinity of the solution [32]. However, it is difficult and computationally expensive to construct and calculate the big Hessian or approximate Hessian for large datasets. Moreover, it may take more iterations to converge if the initialization is not near to the optimal solution.

### PARAFAC2

PARAFAC2 is a useful method for complex high-order tensor analysis. The proposal of PARAFAC2 model can be dated back to the work of Harshman [33]. The PARAFAC2 model is generally written as follows:

$${\mathbf{X}}_{k}=\mathbf{A}{\mathbf{D}}_{k}{\left({\mathbf{B}}_{k}\right)}^{\text{T}}+{\mathbf{E}}_{k}, k=1,\dots ,K \left(s.t. {{\mathbf{B}}_{k}^{\text{T}}\mathbf{B}}_{k}=\mathbf{H} \right) \left(3\right)$$

The definitions of the symbols in Eq. 3 are the same as those in PARAFAC model. The only difference is that each sample now has an individual loading matrix ${\mathbf{B}}_{k}$ instead of the same $\mathbf{B}$ for all samples, and the matrices ${\mathbf{B}}_{k}$ of the shifted mode are constrained to ${{\mathbf{B}}_{k}^{\text{T}}\mathbf{B}}_{k}=\mathbf{H}$ meaning that the profiles of the shifted mode for different samples share the same cross product. In case of GC-MS plant metabolomics data, the evolving ${\mathbf{B}}_{k}$ characterizes the shifted elution profiles for each sample at each run. Together with PARAFAC model, the graphical illustration of PARAFAC2 model is shown on Fig. 3. Kiers et al. developed a direct ALS algorithm which is popular nowadays for fitting PARAFAC2 model [34]. In the direct ALS algorithm, the matrix ${\mathbf{B}}_{k}$ is equally replaced by the product of a $J\times F$ orthogonal matrix ${\mathbf{P}}_{k}$ and a $F\times F$ common matrix $\mathbf{U}$, where $F$ is the number of components in the model and $J$ is the dimension of the shifted mode. PARAFAC2 model is inherently not a strict multi-linear model. Specifically, it does not assume the profiles in a specific mode to keep constant across the samples/slabs in another mode in a third-order tensor. Instead, PARAFAC2 only requires the cross product of the profiles to keep constant across the samples/slabs in another mode [22]. In plant metabolomics analysis, data is most likely not strictly multi-linear for many reasons such as the artifacts, instrument performance, samples status and other environmental factors. By relaxing the strict multi-linearity, the PARAFAC2 model works well for non-strict-multilinear high-order tensor data such as the retention time shifted three-way GC-MS data.

Similar to the PARAFAC model, the solutions of the PARAFAC2 model are also unique under certain conditions [35]. The uniqueness property of PARAFAC2 model is very useful in practice, for example, when PARAFAC2 is applied in curve resolution problem, the chemical profiles of different compounds can be uniquely determined and resolved due to the uniqueness of PARAFAC2 model. This significantly increases the interpretability of the model results and avoids the unnecessary ambiguity.

The PARAFAC2 model is advantageous in many other aspects. For example, compared to the two-way methods (e.g., MCR), the PARAFAC2 fully explores the multi-way structure of the high-order shifted tensor and yields the model with unique solutions and more interpretability. Unlike PARAFAC model, the PARAFAC2 model does not need the cumbersome preprocessing and alignment procedure when analyzing the shifted multi-way GC-MS data [18]. The rotation freedom problem also does not exist in PARAFAC2 model [34]. Moreover, the PARAFAC2 model is practically less sensitive to the shape changes of the factor’s profiles than traditional methods. The invariant cross product requirement in the PARAFAC2 model inherently means the angles of factors profiles in the shifted mode do not change. Therefore, even though the shapes of the factor profiles change a little bit, the data can still be properly analyzed by PARAFAC2 model as long as the angles of the factor’s profiles do not change too much. The popularly used direct PARAFAC2-ALS algorithm can be easily extended to N-way cases, which is potentially useful for solving a wide range of complex applications such as GC-GC-MS or LC-LC-MS plant metabolomics data.

There are still some limitations in PARAFAC2 modeling when it is used for complex plant metabolomics data analysis. The computation of PARAFAC2 model is inherently an NP-hard problem as it is for other tensor decomposition problems [36]. It means the solutions to the hardest problems in NP can be found by answering questions about high-order tensor decomposition problem. One of the obvious difficulties of the NP-hard problem is the occurrence of local minima solutions. A local minimum solution of PARAFAC2 model refers to the inferior solution of the global optimization problem. In other words, the loss function error associated with the local minimum PARAFAC2 model is higher than that associated with the global minimum PARAFAC2 model. The most widely used direct PARAFAC2-ALS algorithm suffers the local minima problem to an extent that cannot be ignored and it has been reported in several applications papers [37, 38]. In some cases, it might happen that the difference of loss function errors between local minimum PARAFAC2 model and global minimum PARAFAC2 model is extremely small, however, the resolved factors profiles of PARAFAC2 models may still have a significant difference. Overlooking the local minima issue may cause wrong and ambiguous expert analysis when the PARAFAC2 model is applied on plant metabolomics data analysis. Even though the widely used PARAFAC2-ALS algorithm is easy to implement and simple to understand, it still has several numerical drawbacks. For example, the efficiency of popular PARAFAC2-ALS algorithm is generally not satisfactory when it is used for analyzing large multi-way plant metabolomics datasets. This is caused not only by the property of the ALS method (only a subset of the estimated variables is changed at one time), but also due to the slow linear convergence rate of PARAFAC2-ALS algorithm. Moreover, the two-factor degeneracy [39] problems have a possibility to occur in the high rank cases when the PARAFAC2-ALS algorithm is employed.

Recently, there has been some work that has been dedicated to tackling the numerical issue of PARAFAC2. Cohen et al. proposed a flexible coupling Non-negativity PARAFAC2 model by relaxing the constraints of the normal PARAFAC2 model and adding the regularization term to the normal loss function of PARAFAC2 model [40]. More recently, Roald et al. developed an algorithm for fitting PARAFAC2 model based on the alternating direction method of multipliers (ADMM) framework [41]. In this method, they added the regularization terms on all the modes in PARAFAC2 model by implementing a splitting scheme on the PARAFAC2 problems. However, the effectiveness of these regularized PARAFAC2 algorithms still needs to be widely tested on different types of multi-way plant metabolomics datasets and the quality of the solutions of these algorithms has to be investigated. Additionally, the coupling effects of these regularized PARAFAC2 algorithms with other numerical optimization techniques (e.g., acceleration techniques) frequently used in tensor decomposition remain further investigations. Faster PARAFAC2 algorithms have been developed in the work of Huiwen et al. [42]. In their work, PARAFAC2 algorithm is significantly accelerated by performing different types of extrapolation enhancements on the estimated factor matrices. The proposed algorithms are recommended to be used for large plant metabolomics data analysis. In order to cope with the local minima issue, new PARAFAC2 algorithms have also been proposed [22]. The new algorithms are validated to be useful for avoiding local minima in the context of PARAFAC2 decomposition.

### Tensor based data fusion

Data fusion is defined as the joint analysis of multiple inter-related datasets that provide complementary views of the same phenomenon [43]. The integration of multi-modal datasets coming from various sources may have the potential of enhancing the systematic understanding, knowledge discovery and information extraction compared to using individual dataset. For example, in plant metabolomics analysis, data measured from NMR and mass spectroscopy-based instrument is complementary, and the joint analysis of the complementary datasets is capable of enhancing the chemical discovery and metabolites identification [44]. In chemometrics, the similar concept was first introduced by Smilde et al. [45]. In tensor-based data fusion, the tensors and the matrices are coupled in the specific modes, and these datasets are decomposed jointly and share the same latent space. The most popular model for performing tensor-based data fusion is the coupled matrix tensor factorization (CMTF) model [46]. In CMTF model, tensor and matrix are jointly decomposed into shared factor and non-shared factors. The uniqueness property of the normal tensor decomposition still remains in CMTF [46]. The ALS algorithm can be employed to calculate CMTF model [47]. In this case, we need to concatenate the two sets of data. In the same manner as in PARAFAC-ALS, all but one of the matrices that we are seeking to estimate is fixed, then a normal ALS procedure can be continued until reaching convergence. The process of a simple tensor coupled matrix data fusion modeling is presented on Fig. 3.

New advances in tensor-based data fusion algorithms have been achieved over the years. Evrim et al. proposed the so-called advanced CMTF(ACMTF) model [48]. The ACMTF models was capable of decomposing both shared and unshared components in the coupled factor(s) and these components can be automatically determined. Mosayebi et al. proposed a new model called correlated CMTF (CCMTF) where the correlation between the shared components of two dataset in the common mode are maximized [49]. The CCMTF model is deemed to alleviate the strict assumption of identical shared components in ACMTF. More recently, a flexible framework for tensor-based data fusion has been proposed by Evrim et al. [50]. Owing to this new framework, a variety of model constraints, loss functions and couplings are possibly to be added into the tensor data fusion models in a flexible way. In addition to the tensor coupled matrix studies, tensor coupled tensor algorithm has also been investigated in recent years. For example, Chatzichristos et al. proposed double coupled tensor decompositions and explored soft and flexible coupling approaches to implement the multi-tensor data fusion modeling [51]. Many advancements have been made over the years, while it still remains challenges in different aspects of tensor-based data fusion. The existence of missing data, heterogeneity of data variables, different types of noise and artifacts, and data uncertainties [43] are all critical problems that we have to face and cope with. All in all, the advancements in tensor-based data fusion methods will definitely enable us to better understand and gain new insights from complex multi-modal/multi-block plant metabolomics datasets.

### Other tensor models

Apart from the widely used PARAFAC, PARAFAC2 and tensor-based data fusion models, there are some other tensor models being developed and used for plant metabolomics applications. Some of the tensor models are developed as a variant of an existed chemometrics model. For example, PARAFAC2×N model is a variant of PARAFAC2 model [52]. It is proposed for high-order tensor data with several shift modes in the data such as GC-GC-MS data. In GC-GC-MS data, it has two modes with retention time shift so that the normal PARAFAC2 model cannot model it. In PARAFAC2×N model, an additional coupling constraint is added to flexible coupling PARAFAC2, and this constraint restricts the descent of the extracted mass spectra calculated from models describing two modes retention time shift. N-way PLS is another typical tensor model which is extended from the conventional two-way PLS model [53]. Inherently, N-way PLS is a regression model that combines the tri-linear decomposition with the Partial Least Square (PLS) model, and it works in a manner that tries to find the weight matrices that maximize the covariance between two score matrices. Proper application of N-way PLS is able to increase the prediction performance, yield robust results and improve the interpretability of the model [53].

Many of the tensor models are designed for a specific analytical and numerical purpose. PARAllel profiles with LINear Dependencies (PARALIND) is one of such models that is established for analyzing the multi-linear tensor data with linear dependency factors [54]. By introducing and capitalizing on the dependency matrix defining the inner relationship between the full rank and the rank-deficiency components, the linear dependent factors profiles can be successfully resolved by the PARALIND model. The advantages of PARALIND model make it useful for a wide variety of complex applications. For example, it is possible to employ PARALIND model to resolve the co-eluted peaks in the aligned plant metabolomics GC-MS or LC-MS data. It has been shown that PARALIND can also deal with the linear dependency of the factor profiles in more than one mode [54]. More recently, a new tensor model called PARAFAC Applied to Shift Invariant Amplitude Spectra (PARASIAS) has been proposed for analyzing shifted multi-way data [55]. The PARASIAS model accelerates the complex shifted tensor analysis by combining spectral transformation and the PARAFAC modeling, which provides new insights on the future investigations for efficient tensor models. Based on PARASIAS model, Poul et al. established a shift-invariant tri-linearity model (SIT) for improving chromatography coupled mass spectrometry data analysis [56]. By adding a flexible tri-linearity implementation into the model, SIT model is able to further accelerate the shifted tensor decomposition and make the implementation of constraints on all shifted modes possible. The new progress in tensor models provides new tools and methods for the analysis of plant metabolomics data, which will greatly promote the further development of plant metabolomics research.

## Applications of tensor models in plant metabolomics

### Plant genetic mutant and phenotyping

Chromatographic metabolomics has been widely used to perform plants genetic mutant and phenotyping studies. As a powerful tool, tensor methods play an important role in understanding the importance of metabolites traits and associated genetic factors. Khakimov et al. explored the seed phenotyping of barley by using PARAFAC2 model on multi-way metabolomics data [57]. The relations between metabolite patterns of barley seed and genotype and growth temperature were revealed. For example, they found that the increase in proteins with rich essential amino acid lysine is caused by the mutation gene in lys3 barley seed. Similar research has been conducted on other plants. Porter et al. investigated the metabolites related to the biosynthetic pathways of indole-3-acetic acid in maize seedling [58]. They employed PARAFAC to model and reveal the metabolite pattern of both mutant-type and wild-type maize seedlings. Another study on cassava focused on identifying the genes regulating the production of specific metabolites by using tensor methods [59]. Specifically, they identified a gene as a catalyst in the synthesis of Linamarin metabolite in cassava by combining PARAFAC modeling and LARS regression. The results were critical for further understanding the systematic relation between genes and metabolites controlling in cassava plant.

Tensor methods provide new insights for metabolites characterization-based plant phenotyping study. PARAFAC2 modeling coupled with PLS-DA was capable of achieving high precision classification of the wild-type and genetically engineered poplars with a success rate more than 99% [60]. By characterizing the small metabolites and its tiny change, PARAFAC2 based method was recommended to be an efficient and promising way for poplars classification. Moreover, the practical applications indicate that tensor methods have strong ability and advantages in plant metabolites characterization. PARAFAC based extraction protocols were validated to extract greater varieties and amounts of metabolites from Erythrina speciosa Andrews leaves compared to the traditional methods [61]. The new method was expected to characterize the chemical fingerprints with high quality in natural products. Modeling efficiency is another concern regarding to large scale plant metabolites characterization. The application of PARASIAS model on barley data indicates that efficient characterization of large-scale plant metabolomics data is possible [55]. This new tensor tool will greatly contribute to the metabolome-wide analysis and the integration analysis of large multi-omics data. Recently, specific analytical challenge of plant metabolites characterization has drawn plant scientist’s attention. For example, a non-negativity PARAFAC2 based work flow has been designed and applied on plant tissue samples in order to improve the resolution of co-eluted peaks in plant metabolomics analysis [62]. The new proposal was validated to be a favorable choice for characterizing complex co-eluted metabolites peaks.

### Plant diseases and resistance

Plant disease and resistance are the eternal research themes of plant science. Investigating plant disease from a metabolomics point of view is important for understanding the complex mechanism of plant disease. Hantao et al. analyzed the volatile metabolites of hybrids of Eucalyptus globulus to determine the Eucalyptus samples susceptibility to rust disease [63]. They combined PARAFAC model and Fisher ration analysis to investigate the correlation between chromatographic chemical profiles and resistance against Eucalyptus rust. From this, the susceptible plants were discriminated successfully. In another study, a PARAFAC and LC-MS based plant disease diagnosis method for Eucalyptus globulus was developed [64]. Specifically, they performed the distinction of healthy samples and non-healthy samples and identified the metabolites related to the biotic stress by using the PARAFAC scores and loadings. The new method was deemed to provide new insights into the analysis of plant disease and defense mechanism. The control of plant diseases is very important for plant growth. Tensor based metabolomics technology can be useful to this field. As an example, Bordagaray et al. used PARALIND model to resolve metabolites with high similarity spectra from the mixture of plant fungicides [65]. The complex chemicals in the mixture were successfully resolved, which was very important for understanding the composition of plant fungicides.

The investigations on plant resistance against to insects is vital for protecting plant growth. Tensor methods have been applied in plant metabolomics to help understand the plant resistance mechanism. Khakimov et al. employed PARAFAC2 model on the LC-MS metabolomics data of Barbarea vulgaris plants [66]. By combining tensor method with PLS and correlation analysis, five unknown saponin-like compounds correlated with the resistance of plants against to insect herbivore were successfully found, and these compounds have not been detected using traditional chemometrics tools before. Similar research can be found in the recent work. Gonzalez et al. used PARAFAC2 modeling on GC-MS metabolomics data to investigate the effect of endophytic colonization by the entomopathogenic fungus Beauveria bassiana on melon and cotton plants [67]. By doing so, they systematically explored the plant defense responses to insect-pathogenic fungi which plays a key role in integrated pest management systems. Jan et al. presented that the amino acid residues at position 121 and 735 accounted for the production ratio of the resistance chemicals against to insect herbivores [68]. PARAFAC2 modeling on GC-MS barbarea vulgaris leaves data was performed in the study from which the role of enzymes as important mediators of metabolic plasticity throughout plant evolution were revealed. Hence it is evident from the above studies that the tensor methods-based metabolomics technology is currently taking effects on investigating the resistance of plants against to insects and the plant growth protection.

### Plant pharmacology and nutrition

The pharmacological components analysis of plants is an important topic that cannot be ignored in plant science research, as well as in plant industry. However, due to the complexity of medicinal and edible plant systems, accurate characterization of their pharmacological components is not straightforward. Metabolomics technology provides an inspiring solution for the analysis and quality assessment of plant pharmacological components. In particular, the coupling of tensor methods and metabolomics technology further promotes the exploration on this issue. Schmidt et al. applied PARAFAC model on the aligned HPLC metabolomics data generated from Hypericum perforatum used for producing herbal preparations [69]. The differences in composition between individuals were successfully detected. The established workflow provided a tool for unsupervised and unbiased assessment of the composition of herbal preparations, being important for evaluation of plant pharmacological activity. Recently, Turova et al. have also proposed a PARAFAC-based algorithm for herbal extracts identification [70]. The proposed method was applied on HPLC-MS data generated from a variety of plants extracts such as Glycyrrhiza glabra and Panax ginseng dried root. The new protocol was validated to be capable of robustly identifying the critical metabolites composition and thus being a robust tool for quality control of plant pharmacological components. A variety of similar research on this topic can be found in the recent work [71–73]. These studies provide new tensor insights with a forward-looking perspective for robust, reliable and rapid pharmacological component analysis and quality evaluation of plants.

The coupling of tensor modeling and nutrient composition analysis can be observed from the plant metabolomics literature. Khakimov et al. conducted a detailed nutrient value analysis of the main northern European cereal crop plants by comparing the metabolites profiles of different cereal crop plants [74]. Compared to the traditional tools, the proposed PARAFAC2 based protocol was reported to provide an efficient and high throughput analysis of the cereal metabolites and improve the detection of conjugated phenolics. For a systematic knowledge and applications about cereal metabolomics and nutrient composition analysis, we refer to the review [75]. The nutrition analysis of plant and its related issues in the context of metabolomics is gaining more and more attention from scientific community [76, 77]. Even though tensor-based method is taking its power in plant nutrients composition analysis, its potential is far from reached and more opportunities for the wide applications are expected in the future.

### Plant products characterization and evaluation

Plants provide values to humans and society in the form of plant products in many cases. Along the years, the study of plant products has attracted increasing interests from both plant scientists and analytical scientists. Favilla et al. employed the discriminant version of N-way PLS-DA model (NPLS-DA) and Variable Importance in Projection (VIP) method to efficiently evaluate the authenticity of extra virgin olive oils [78]. The tensor method was validated to provide a favorable tool for robust olive oils assessment. Silvestri et al. used PARAFAC based method to jointly analyze the HPLC, NMR and fluorescence datasets of Lambrusco grape wine samples [79]. A data fusion protocol was established for well characterizing the phenolic metabolites of Lambrusco grape wine. Schenker et al. optimized the tensor-based data fusion method CMTF and used it to analyze joint data from multiple metabolomics platform such as NMR and LC-MS, their results indicated that tensor-based data fusion model enhanced the metabolites discovery from complex plant products mixture [50]. Similar research on tensor-based data fusion applications can be observed from other work [44]. Efficient and robust characterization of large-scale metabolites of plant products is important for fulfilling the needs of automated plant production process. Recently, Schneide et al. has applied SIT model on GC-MS apple wine data [56]. The tensor method has a pretty high efficiency for modeling large scale GC-MS plant metabolomics data. Compared to the state-of-the-art curve resolution method, the SIT model was 60 times faster in the best case. SIT model will definitely advance the automated online metabolites analysis of plant products in the future. As expected, PARAFAC and PARAFAC2 based plant metabolomics methods are very popular in the quality evaluation and classification analysis of plant products. Their applications cover a wide variety of plant products such as corn oil, coffee, olive oil and grape wine etc. [80–85]. Tensor methods have been verified on these applications for being able to provide clearer identification and assignments of metabolites, higher quality chromatographic fingerprints, more robust modeling results and more reliable quality assessments compared to the traditional chemometrics tools. The details of typical applications of various tensor models in plant metabolomics analysis are listed in Table 1.

**Table 1:** The typical applications of tensor models in plant metabolomics investigations

Category	Plants	Tensor models	Instruments	Analytical purpose	Reference
Plant genetic mutant and phenotyping	Barley	PARAFAC2	GC-MS	improve metabolites identification	[22]
	Tobacco	PARAFAC2	GC-MS	enhance metabolites resolution	[38]
	Barley	PARASIAS	GC-MS	plant phenotyping and metabolites characterization	[55]
	Barley	PARAFAC2	GC-MS	phenotype, genetic and environmental analysis	[57]
	Maize	PARAFAC	2D-LC-DAD	genetic mutant and metabolites analysis	[58]
	Cassava	PARAFAC	LC-MS	identify genes for regulating metabolites	[59]
	Poplars	PARAFAC2	Py-GC-MS	plant phenotyping and classification	[60]
	Erythrina speciosa Andrews leaves	PARAFAC	HPLC-DAD	plant phenotyping and metabolites characterization	[61]
	Lupinus angustifolius	Non-negativity PARAFAC2	UHPLC-HRMSE	metabolites characterization	[62]
Plant diseases and resistance	Eucalyptus globulus	PARAFAC	GC-GC-qMS	plant disease susceptibility	[63]
	Eucalyptus globulus	PARAFAC	LC-MS	plant disease diagnosis	[64]
	Plant related products	PARALIND	HPLC-DAD	fungicides composition	[65]
	Barbarea vulgaris	PARAFAC2	LC-MS	plant resistance analysis	[66]
	Melon and cotton	PARAFAC2	GC-MS	plant resistance analysis	[67]
	Barbararea vulgaris	PARAFAC2	GC-MS	plant resistance analysis	[68]
Plant pharmacology and nutrition	Hypericum perforatum	PARAFAC	HPLC-DAD	herbal preparations composition analysis	[69]
	Glycyrrhiza glabra and Panax ginseng dried root etc.	PARAFAC	HPLC-MS	quality control of plant pharmacological components	[70]
	Quinoa	PARAFAC2	GC-MS and LC-MS	pharmacological components analysis	[71]
	Cyperi rhizoma	PARAFAC	LC-MS	analysis on active ingredients in Chinese herbs	[72]
	Teucrium polium	PARAFAC	GC-MS	composition characterization	[73]
	Cereal crop	PARAFAC2	GC-MS	nutrient value analysis	[74]
	Tropical fruits	PARAFAC2	GC-MS	nutrition and quality evaluation	[77]
Plant products characterization and evaluation	Apple wine	PARAFAC2	GC-MS	enhance metabolites resolution	[42]
	Apple wine	SIT	GC-MS	accelerate the metabolites resolution	[56]
	Olive oils	NPLS-DA	GC-MS	authentication analysis	[78]
	Lambrusco grape wine	PARAFAC	HPLC-MS	based data fusion classification	[79]
	Mixture	CMTF	LC-MS	enhance metabolites discovery	[50]
	Corn oil	PARAFAC and PARAFAC2	LC-LC-MS	enhance metabolites discovery	[80]
	Grape wine	PARAFAC2	HS-SPME-GC-MS	regionality and quality analysis	[81]
	Olive oil	PARAFAC2	GC-MS	quality classification	[82]
	Cottonseed oils	PARAFAC2	GC-MS	quality classification	[83]
	Grape wine	PARAFAC2	GC-MS	quality classification	[84]
	Coffee	PARAFAC	HPLC-DAD	metabolites identification	[85]

## Conclusions

The rapid development of metabolomics technology has profoundly affected the field of plant science. The advances in chemometrics provide the key tools for data analysis and processing in plant metabolomics research. Among them, advanced chemometrics tools represented by tensor analysis methods have undoubtedly further promoted the automated and intelligent process of plant metabolomics analysis. Although the tensor analysis method has been widely used in plant metabolomics research, its potential is far from being reached. The synergy between tensor analysis methods and high-throughput metabolomics data analysis summarized in this review is just the tip of the iceberg. In the future, there will be more opportunities for tensor-based advanced chemometrics methods in solving complex plant metabolomic analysis problems. More advanced tensor chemometrics tools will continue to emerge in order to continuously adapt to the increasing needs of plant metabolomics research during its evolution process.

## Data Availability

The materials used to support the findings of this study can be made available by the corresponding author upon request.
